# The importance of skin–to–skin contact for early initiation of breastfeeding in Nigeria and Bangladesh

**DOI:** 10.7189/jogh.07.020505

**Published:** 2017-12

**Authors:** Kavita Singh, Shane M Khan, Liliana Carvajal–Aguirre, Paul Brodish, Agbessi Amouzou, Allisyn Moran

**Affiliations:** 1 MEASURE Evaluation/Carolina Population Center, University of North Carolina at Chapel Hill, Chapel Hill, North Carolina, USA; 2 Department of Maternal and Child Health, Gillings School of Global Public Health, University of North Carolina at Chapel Hill, Chapel Hill, North Carolina, USA; 3 Data and Analytics, Division of Data, Research and Policy, UNICEF, New York, New York, USA; 4 Department of International Health, Johns Hopkins Bloomberg School of Public Health, Baltimore, Maryland, USA; 5 Global Health Fellows Program II, United States Agency for International Development (USAID), Washington, D.C., USA

## Abstract

**Background:**

Skin–to–skin contact (SSC) between mother and newborn offers numerous protective effects, however it is an intervention that has been under–utilized. Our objectives are to understand which newborns in Bangladesh and Nigeria receive SSC and whether SSC is associated with the early initiation of breastfeeding.

**Methods:**

Demographic and Health Survey (DHS) data were used to study the characteristics of newborns receiving SSC for non–facility births in Nigeria (DHS 2013) and for both facility and non–facility births in Bangladesh (DHS 2014). Multivariable logistic regression was used to study the association between SSC and early initiation of breastfeeding after controlling for key socio–demographic, maternal and newborn–related factors.

**Results:**

Only 10% of newborns in Nigeria and 26% of newborns in Bangladesh received SSC. In the regression models, SSC was significantly associated with the early initiation of breastfeeding in both countries (OR = 1.42, 95% CI 1.15–1.76 for Nigeria; OR = 1.27, 95% CI 1.04–1.55, for Bangladesh). Findings from the regression analysis for Bangladesh revealed that newborns born by Cesarean section had a 67% lower odds of early initiation of breastfeeding than those born by normal delivery (OR = 0.33, 95% CI 0.26–0.43). Also in Bangladesh newborns born in a health facility had a 30% lower odds of early initiation of breastfeeding than those born in non–facility environments (OR = 0.70, 95% CI 0.53–0.92). Early initiation of breastfeeding was significantly associated with parity, urban residence and wealth in Nigeria. Geographic area was significant in the regression analyses for both Bangladesh and Nigeria.

**Conclusions:**

Coverage of SSC is very low in the two countries, despite its benefits for newborns without complications. SSC has the potential to save newborn lives. There is a need to prioritize training of health providers on the implementation of essential newborn care including SSC. Community engagement is also needed to ensure that all women and their families regardless of residence, socio–economic status, place or type of delivery, understand the benefits of SSC and early initiation of breastfeeding.

Globally there were an estimated 5.9 million deaths to children under–five in the year 2015, and 45% of these deaths occurred during the neonatal period, the first month of life [1]. While there have been substantial reductions in under–five mortality, reductions in neonatal mortality have been less pronounced. From 1990 to 2015 under–five mortality fell by 52% compared to 42% for neonatal mortality [1]. The main causes of under–five mortality are now prematurity, pneumonia; and intrapartum–related conditions including birth asphyxia [2]. Notably two of the top three main causes of death occur either exclusively in the neonatal period (birth asphyxia) or mostly in the neonatal period (prematurity), while pneumonia is a cause of death for both neonates and children 1–59 months. The global health community has provided specific recommendations on essential newborn care (ENC), which refers to care provided to the newborn within the first moments to days of life [3]. Components of ENC include thermal care, early and exclusive breastfeeding, appropriate cord care and monitoring/early treatment for low birth weight or sick newborns. ENC is intended to enable countries to protect newborns through the implementation of simple, yet, life–saving interventions.

The ENC interventions focused on thermal care are intended to ensure that newborns do not develop hypothermia (state of being too cold) or hyperthermia (state of being too hot). Newborns regulate temperature less effectively and lose heat more easily compared to adults. These issues are intensified for low birth weight and premature newborns [4]. Interventions for thermal care include immediate drying, delayed bathing, head covering and skin–to–skin contact (SSC), which is the placement of a naked newborn baby prone on the mother’s bare chest soon after birth [5]. The mother and baby may be covered loosely with a blanket or cloth, preferable pre–warmed [6]. The newborn baby should remain in that position until the end of the first successful breastfeeding [5,7]. In addition to providing warmth, SSC also has numerous other benefits including improved attachment between mother and newborn [8–10] and the reduction of infant stress [5,11,12]. A Cochrane Review of randomized trials including mother–baby dyads, found that SSC at birth was associated with breastfeeding at one to four months post–birth with a risk ratio of 1.25. A total of 13 trials, composing of 702 mother–baby dyads, were included in the review for the breastfeeding outcomes [5]. Despite the benefits and the simplicity of SSC as a natural intervention, over time it became common practice to separate newborns from their mothers after delivery often due to routine procedures [5,13–5]. In 2012, the American Academy of Pediatrics stated that many maternal and newborn assessments can be done during SSC or can be delayed until after the critical SSC period, so long as the mother and newborn do not have any complications [16].

There is an increased focus on newborn health interventions among the global health community and recently some large–scale household surveys have included measures of such interventions. The use of population–based data are important, as it can provide some indication of how well a country is implementing SSC in real–life settings. Data on SSC are only currently available from a few household surveys, most recently the Nigeria Demographic and Health Survey (DHS) 2013 [17] and the Bangladesh DHS 2014 [18]. These two countries together accounted for 12% of the world’s neonatal deaths [1].

The first objective of this analysis is to assess the level of practice of SSC in Nigeria and Bangladesh and to examine the characteristics of newborns who are receiving SSC. Understanding SSC by key factors is essential in efforts to improve coverage of this intervention. The second objective is to determine whether SSC is associated with the early initiation of breastfeeding, defined as breastfeeding within the first hour of life. Early initiation of breastfeeding is an important outcome to study for several reasons. First milk or colostrum is rich in protective factors including antibodies and vitamin A, and early breastfeeding is a pivotal step towards longer–term and exclusive breastfeeding [19]. Trials in Nepal and Ghana have found that early initiation of breastfeeding could prevent 19% and 22%, respectively, of neonatal deaths [20,21].

## Methods

### Data sources

We used data from the 2013 Nigeria DHS and the 2014 Bangladesh DHS. The DHS are a source of nationally representative data for monitoring socio–economic and health indicators at the population level. In sampled households women age 15–49 are eligible to participate, but in some countries, such as Bangladesh, only ever–married women 15–49 are eligible to participate. The sample is based on a stratified two–stage cluster design. The first stage is the sample enumeration area (SEA), and the second stage is a list of households from each SEA. The samples are representative at the national, urban/rural residence and regional levels. For both Nigeria and Bangladesh we restricted our sample to women with a live birth. For Nigeria the question on SSC was asked of the most recent birth in the past five years for non–facility births only. For Bangladesh the question on SSC was asked of the most recent birth in the past three years for both facility and non–facility births. (The specific questions are described in the section on the key independent variable.) The sample sizes were 11 966 mother–newborn pairs in Nigeria and 4444 mother–newborn pairs in Bangladesh.

### Outcome variable

The dependent variable was early initiation of breastfeeding, defined as breastfeeding within one hour of birth. The question was worded the same in both Bangladesh and Nigeria: *How long after birth did you first put (NAME) to the breast?*

The variable was coded as “0” if more than 1 hour and “1” if one hour or less.

### Key independent variable

The key independent variable was a dichotomous indicator of SSC for the most recent birth in the past three (Bangladesh) or five (Nigeria) years. The questions were slightly different between the two surveys as shown below.

Nigeria (non–facility births only): *Was (Name) placed on your belly/breast before delivery of the placenta?*

Bangladesh (facility and non–facility births): *After the birth was (Name) put directly on the bare skin of your chest?*

Another difference is that in Bangladesh the interviewers were trained to show the respondent a picture of the SSC position.

### Control variables

Several maternal health variables were studied including mother’s age in years by the following age groups: 15–19, 20–24, 25–34, 35+; education (none, primary, secondary or higher); current marital status (married, not married) and parity (1, 2–3, 4+). The analysis included two socio–economic variables – urban residence and wealth quintile and a demographic variable–subnational region of residence (which were zones for Nigeria and divisions for Bangladesh). Several delivery–rated factors were studied including Cesarean delivery (yes/no: Bangladesh only); facility delivery (yes/no Bangladesh only; facility vs non–facility) and type of delivery attendant [skilled birth attendant (SBA) vs unskilled birth attendant]. SBA was defined as a doctor, nurse or midwife in accordance with the global definition. We included mother’s perceptions of her baby’s birthweight (small, average, large, large) to understand any differentials in SSC by perceived size of the baby.

### Analysis

The analysis was carried out separately for each country. Bivariate analyses (using Pearson χ^2^ test) compared several control variables for mothers reporting SSC immediately after birth to those who did not. In multivariate logistic regressions, early breastfeeding was regressed on SSC, controlling for all of the maternal and infant–related variables mentioned in the section above.

## RESULTS

**Table 1** shows column percentages of SSC by key characteristics of the mother and newborn in Nigeria. Only about 10% of mothers (1217/12 265) reported SSC, and there was little difference between mothers whose newborns received SSC and mothers whose newborns did not receive SSC. However, there was one significant difference. Newborns who were perceived to be large were significantly more likely to experience SSC than smaller newborns. About 55% of the newborns who received SSC were perceived to be large, compared to 29% and 16% for those perceived to be average and small, respectively.

**Table 1:** Skin-to-skin contact by key characteristics in Nigeria: Last non–facility birth in past five years (survey–weighted column percentages and counts)

	Skin–to–skin contact						
	**Yes**		**No**		**Total**		***P***
	**10%**	**(n = 1217)**	**90%**	**(n = 11 048)**	**100.0%**	**(N = 12 265)**	
**Mother’s age:**	**%**	**n**	**%**	**n**	**%**	**n**	
15–19	7.9	96	7.7	850	7.7	946	
20–24	20.3	248	20.9	2305	20.8	2551	
25–34	45.2	551	44.9	4964	45.0	5515	
35–49	26.6	323	26.5	2929	26.5	3249	
**Parity:**							
1	13.8	168	14.6	1616	14.6	1784	
2–3	26.4	321	29.2	3223	28.9	3545	
4+	59.8	728	56.2	6204	56.5	6932	
**Mother’s education:**							
None	70.3	856	68.2	7538	68.5	8394	
Primary	17.8	217	17.0	1877	17.1	2093	
Secondary+	11.8	144	14.8	1633	14.5	1774	
**Zone:**							
North Central	16.3	198	11.1	1227	11.6	1425	
North East	33.8	412	20.4	2253	21.7	2665	
North West	42.9	523	52.6	5809	51.6	6332	
South East	1.2	14	2.7	297	2.5	312	
South South	4.1	50	7.3	806	7.0	856	
South West	1.7	21	5.9	651	5.5	672	
**Residence:**							
Urban	18.7	228	20.4	2263	20.3	2486	
Rural	81.3	990	79.6	8785	79.7	9775	
**Wealth:**							
Poorest	38.6	470	34.8	3843	35.2	4313	
Second	27.2	331	29.8	3294	29.6	3624	
Middle	18.2	221	18.6	2057	18.6	2279	
Fourth	11.5	140	11.8	1300	11.7	1438	
Richest	4.6	56	5.0	553	4.9	606	
**Estimated size at birth:**							
Small	16.0	193	18.0	1973	17.8	2167	
Average	29.4	355	42.2	4630	40.9	4985	
Large	54.7	662	39.9	4378	41.3	5040	< 0.001
**Attendant at delivery:**							
Doctor/midwife/Nurse	5.5	67	3.8	417	4.0	484	
Unskilled provider/friend/family member/other	94.5	1141	96.2	10 588	96.0	11 729	

**Table 2** shows column percentages of SSC by key characteristics for the Bangladesh sample. Overall, about 26% of mothers (1210/4586) reported SSC with their newborn, and once again there was little difference between mothers whose newborns received SSC vs those that did not. The only significant finding was that newborns of parity 2–3 were significantly more likely to have experienced SSC with their mothers compared to newborns of parity one and higher parity. Of the newborns receiving SSC 50% were of parity 2–3 compared to 39% for newborns of parity one and 11% for newborns of parity 4 and higher. This finding, however was only significant at *P* < 0.10.

**Table 2:** Skin–to–skin contact by key characteristics in Bangladesh: last birth in past three years (survey–weighted column percentages and counts)

	Skin–to–skin contact						
	**Yes**		**No**		**Total**		***P***
	**26%**	**(n = 1210)**	**84%**	**(n = 3376)**	**100.0%**	**(N = 4586)**	
**Mother’s age:**	**%**	**n**	**%**	**n**	**%**	**n**	
15–19	19.7	238	21.3	718	20.9	957	
20–24	33.2	401	33.7	1138	33.6	1540	
25–34	41.9	506	38.7	1305	39.5	1812	
35–49	5.2	63	6.3	214	6.0	277	
**Parity:**							
1	38.5	465	40.1	1352	39.6	1818	
2–3	50.3	609	45.1	1524	46.5	2132	
4+	11.2	136	14.8	500	13.9	636	< 0.1
**Mother’s education:**							
None	11.8	143	15.1	508	14.2	651	
Primary	28.6	346	27.7	936	28.0	1283	
Secondary+	59.6	721	57.2	1931	57.8	2652	
**Division:**							
Barisal	6.1	74	5.7	192	5.8	267	
Chittagong	17.5	212	23.4	791	21.9	1003	
Dhaka	40.2	486	33.6	1135	35.4	1621	
Khulna	8.6	104	7.7	259	7.9	363	
Rajshahi	9.0	109	10.5	353	10.1	462	
Rangpur	9.5	114	9.8	332	9.7	446	
Sylhet	9.1	110	9.3	314	9.2	424	
**Residence:**							
Urban	26.2	317	26.1	881	26.1	1198	
Rural	73.8	893	73.9	2495	73.9	3388	
**Wealth:**							
Poorest	22.1	268	21.6	730	21.8	997	
Second	17.6	213	19.5	659	19.0	872	
Middle	19.7	238	18.7	632	19.0	870	
Fourth	22.0	266	20.0	675	20.5	942	
Richest	18.6	225	20.2	680	19.7	905	
**Estimated size at birth:**							
Small	22.7	275	18.9	637	19.9	912	
Average	65.5	793	67.9	2291	67.3	3084	
Large	11.7	142	13.2	447	12.8	589	
**Delivery location:**							
Home	58.0	701	62.8	2121	61.6	2822	
Health facility	38.6	466	34.8	1175	35.8	1641	
Public	15.5*	187	12.1	409	13.0	596	
Private	23.1*	279	22.7	767	22.8	1046	
Other	3.4	41	2.4	80	2.6	121	
**Attendant at delivery:**							
Doctor/midwife/nurse	33.3	402	31.7	1064	32.1	1465	
Unskilled provider/friend/family member/ other	66.7	803	68.3	2293	67.9	3096	
**Caesarean delivery:**							
Yes	23.3	281	24.2	817	24.0	1099	
No	76.7	928	75.8	2558	76.0	3486	

*Percentages of total deliveries.

**Table 3** shows results from regression analyses of early breastfeeding on SSC, controlling for key characteristics of mothers and newborns. In both Nigeria and Bangladesh, SSC was associated with significantly increased odds of early breastfeeding, controlling for all other variables in the models. The odds of early breastfeeding was 42% for newborns receiving SSC in Nigeria and 27% for newborns receiving SSC in Bangladesh (odds ratio (OR) = 1.42, 95% confidence interval (CI) 1.15–1.76; OR = 1.27, 95% CI 1.04–1.55, respectively). Also, in Nigeria several maternal demographic variables were associated with increased odds of early breastfeeding. Newborns of parity 2 or 3 had a 23% increased odds of SSC than newborns of parity 1 (OR = 1.23, 95% CI 1.02–1.50). Compared to North Central, the referent region, the odds of early breastfeeding were significantly lower for newborns in North West, South East and Southwest. Newborns from wealthier households were significantly more likely to experience early breastfeeding than newborns from the very poorest households. The odds ranged from 30% to 59% depending on the specific wealth quintile.

**Table 3:** Survey–weighted logistic regression analysis of early breastfeeding on skin–to–skin contact for the most recent birth, controlling for maternal and infant characteristics

Characteristic	Nigeria (N = 11 419)			Bangladesh (N = 4262)		
*Predictor variable*	**OR**	**95% CI**		**OR**	**95% CI**	
**Skin–to–skin contact**	1.42‡	1.15, 1.76		1.27*	1.04, 1.55	
*Delivery characteristics*						
**Facility delivery:**						
No	NA			1.00		
Yes				0.70*	0.53, 0.92	
**Caesarean delivery:**						
No	NA			1.00		
Yes				0.33‡	0.26, 0.43	
**Estimated size at birth:**						
Small	1.00			1.00		
Average	1.10	0.94, 1.28		1.06	0.87, 1.31	
Large	1.08	0.92, 1.26		0.96	0.68, 1.35	
**Attendant at delivery:**						
Unskilled provider/friend/family member/other	1.00			1.00		
Doctor/midwife/nurse	1.03	(0.78, 1.37)		1.43	0.98, 2.09	
*Maternal demographic characteristics*						
**Age:**						
15–19	1.00			1.00		
20–24	1.07	0.86, 1.32		1.14	0.92, 1.42	
25–34	1.15	0.92, 1.45		0.94	0.73, 1.20	
35–49	1.23	1.02, 1.50		0.74	0.44, 1.25	
**Parity:**						
1	1.00			1.00		
2–3	1.23*	1.02, 1.50		1.08	0.90, 1.31	
4+	1.18	0.95, 1.47		1.04	0.75, 1.43	
**Zone/Division:**						
Nigeria Bangladesh						
North Central Barisal	1.00			1.00		
North East Chittago	1.21	0.95, 1.55		0.83	0.60, 1.14	
North West Dhaka	0.58‡	0.46, 0.73		1.15	0.80, 1.64	
South East Khulna	0.45†	0.28, 0.71		0.81	0.57, 1.14	
South South Rajshahi	0.92	0.71, 1.18		1.22	0.88, 1.70	
South West Rangpur	0.40‡	0.28, 0.57		1.50*	1.03, 2.17	
Sylhet				1.42*	(1.02, 1.96)	
**Residence:**						
Rural	1.00			1.00		
Urban	1.91‡	1.49, 2.43		0.85	0.67, 1.08	
**Education:**						
None	1.00			1.00		
Primary	1.07	0.91, 1.26		0.90	0.66, 1.22	
Secondary+	1.05	0.86, 1.28		0.86	0.65, 1.13	
**Wealth:**						
Poorest	1.00			1.00		
Second	1.30‡	1.08, 1.58		0.85	0.66, 1.10	
Middle	1.37*	1.08, 1.75		0.93	0.70, 1.26	
Fourth	1.59†	1.20, 2.11		1.13	0.84, 1.52	
Richest	1.47*	1.00, 2.16		1.06	0.75, 1.51	
**Marital status:**						
Not married	1.00			1.00		
Married	0.96	0.76, 1.21		1.00	0.41, 2.44	

*P < 0.05.

†*P* < 0.01.

‡*P* < 0.001.

The results for Bangladesh indicated that Cesarean delivery was associated with a 67% lower odds of early breastfeeding (OR = 0.33, 95% CI 0.26–0.43), and facility delivery was associated with a 30% lower odds of early breastfeeding (OR = 0.70, 95% CI 0.53–0.92). There were also two significant effects for division. Compared to the referent division, Barisal, residence in Rangpur was associated with a 50% higher odds of early breastfeeding (OR = 1.50, 95% CI 1.03–2.17) and in Sylhet with a 42% higher odds of early breastfeeding (OR = 1.42, 95% CI 1.02–1.96).

## DISCUSSION

SSC is a natural intervention with numerous benefits, yet it is under–practiced as many mothers and newborns are separated after birth often due to routine procedures [5,13–15]. SSC has been studied as part of trials, but this is the first analysis to use population–level data to look at coverage and factors related to coverage. Associations between SSC with the early initiation of breastfeeding were also studied given the numerous benefits of early breastfeeding, including reduced infant mortality [20,21]. SSC is also considered step 4 of 10 Steps to Successfully Breastfeeding promoted by the Baby Friendly Hospital Initiative [22].

In our study, we found low coverage of SSC in both Nigeria (10%) and Bangladesh (26%) and few differences between newborns receiving SSC and those not receiving SSC. Though an uncommon intervention, in the regression models SSC was significantly associated with early initiation of breastfeeding in both Nigeria and Bangladesh. Thus, our results support findings from trials indicating that SSC is associated with improved breastfeeding outcomes [5] and specifically with early breastfeeding.

Another key finding from the first regression models was that in Bangladesh newborns born in a health facility were less likely to experience early breastfeeding than those born in non–facility environments. There are several plausible explanations for this finding. Some of the mothers and babies may have gone to the facility because of a complication, and this complication may have required separation beyond one hour. A second possible explanation involves health facility procedures, which often require the immediate separation of mother and newborns, which in turn prevents both SSC and early breastfeeding. Many maternal and newborn assessments can actually be done during SSC or can be delayed until after the critical SSC period, so long as the mother and newborn do not have any complications [16,23–25]. Findings from Bangladesh revealed that newborns of mothers who had a Cesarean section were significantly less likely to be breastfed early. Other studies have found the same results [26,27], but there is an increasing recognition that health facilities must implement protocols that allow mothers who have a Cesarean section to breastfeed early. The Baby Friendly Hospital Initiative recommends that SSC can actually begin in the operating theater (after a Cesarean section) when the mother is alert [22,25].

Skilled delivery in a health facility is promoted as an essential strategy to improve both maternal and newborn health [28,29]. The ideal situation would be for women to deliver in a health facility with a SBA who can oversee SSC, as mother and baby should be monitored in case any complication or safety concerns should arise [30].

Other significant findings from the regression analyses are also worthy to note. In both countries there were some differences by zone or division. In Nigeria the northern zones are generally poorer than the southern zones. However early breastfeeding was less common in the southern zones than in the North Central zone. A study by Berde and Yalcin also found variation in the early initiation of breastfeeding by zone [31]. Our results from Bangladesh indicated that early breastfeeding was more common in Rangpur and Sylhet, the poorest divisions, compared to Barisal. A systematic review of early breastfeeding in South Asia also found geographic differences in the early initiation of breastfeeding within countries and also highlighted the influence of traditional practices, which may vary within countries [32]. Another systematic review highlighted the importance of the knowledge and beliefs of family members, particularly for women who deliver at home [33]. A study from Nepal attributed variations in early breastfeeding to many factors including socioeconomic factors, geographic terrain and the availability of formula [34]. Our findings from Nigeria suggested that wealthier and urban women as well as those of parity 2 or 3, had increased odds of having their newborns initiate breastfeeding early. These women may have greater knowledge than their counterparts and some previous experience with breastfeeding. Perceived size at birth was not significant in our regression analyses, but other studies have found that low birthweight and premature newborns are less likely to be breastfed early than normal weight and term newborns [32,33]. Taken together, these comprehensive findings suggest that there is a need to ensure equitable diffusion of knowledge on the importance of early breastfeeding and SSC to all delivery attendants and to all women and families regardless of wealth, parity or geographic residence.

There are several limitations to this analysis. The questions on SSC were not the same for the two countries, and SSC was only asked to women who had a non–facility delivery in Nigeria. More broadly additional work may be needed to validate measures of SSC. A validation study by Blanc et al. 2016 [35] in Kenya found that questions on SSC (newborn placed against mother’s chest after delivery and newborn was naked on skin, not wrapped in towel) did not perform well in terms of individual–level reporting accuracy and population–level accuracy. In this study women’s reports before discharge were compared to direct observation in two hospitals. However, Stanton et al. 2013 [36] found that a question on SSC met the criteria for quality reporting in study in Mozambique, which compared women’s reporting in household surveys (8 to 10 months after delivery) to direct observations. Further work may be needed on exact wording for questions on SSC and for appropriate probes. Another limitation is that the DHS data did not yield complete information on actual birthweight, which is often unknown for newborns who are born at home and not weighed. Also lacking were information on maternal and newborn complications as well as quality of care at the facilities and characteristics of providers. Recall bias could also be an issue in that mothers were asked to recall an event that occurred in a one–hour period. Some of the mothers had births several years (three to five) before the survey.

In terms of program recommendations, training of SBAs on proper thermal care for newborns including SSC is a key step in improving newborn health. The WHO’s Essential Newborn Training Guide includes modules on thermal care including SSC [6]. Manasyan et al. 2011 found this training to be cost–effective for midwives at first level health facilities in Zambia [37]. Trainings will need to be done on a large scale to ensure that all healthy newborns, regardless of delivery type (vaginal vs Cesarean), size or socioeconomic status, receive SSC. At the same time community engagement is needed to enable more mothers and families to learn about the protective effects of SSC. More research is needed on exact timing of initiation of SSC, frequency and duration as well as measures to ensure SSC is as safe as possible [5]. SSC is an intervention with the potential to save newborn lives.

## References

[1] UNICEF. Levels and trends in under-five mortality. Estimates developed by the UN Interagency Group for Child Mortality Estimation. New York: UNICEF; 2015.

[2] WHO. MCEE-WHO methods and data sources for child causes of death 2000-2015. Global Health Estimates Technical Paper WHO/HIS/IER/GHE/2016.1 Geneva: WHO; 2016.

[3] WHO. Every newborn: An action plan to end preventable deaths. Geneva:WHO; 2014.

[4] WHO. Thermal Protection of the Newborn: A practical guide. The Safe Motherhood Initiative. Geneva: WHO; 1997.

[5] Moore ER, Anderson G, Bergman N, Doswell T (2012). Early skin-to-skin contact for mothers and their healthy infants.. Cochrane Database Syst Rev.

[6] WHO. Essential newborn care training module. 2010. Available: http://www.who.int/maternal_child_adolescent/documents/newborncare_course/en/. Accessed: 6 February 2017.

[7] Widström AM, Lilja G, Aaltomaa-Michalias P, Dahllöf A, Lintula M, Nissen E (2011). Newborn behaviour to locate the breast when skin-to-skin: a possible method for enabling early self-regulation.. Acta Paediatr.

[8] Nahidi F, Tavafian SS, Heidarzadeh M, Hajizadeh E, Montazeri A (2014). The Mother-Newborn Skin-to-Skin Contact Questionnaire (MSSCQ): development and psychometric evaluation among Iranian midwives.. BMC Pregnancy Childbirth.

[9] Saastad E, Ahlborg T, Froen JF (2008). Low maternal awareness of fetal movement is associated with small for gestational age infants.. J Midwifery Womens Health.

[10] Mikiel-Kostyra K, Mazur J, Bołtruszko I (2002). Effect of early skin-to-skin contact after delivery on duration of breastfeeding: a prospective cohort study.. Acta Paediatr.

[11] Thukral A, Sankar MJ, Agarwal R, Gupta N, Deorari AK, Paul VK (2012). Early skin-to-skin contact and breastfeeding behaviors in term neonates: A randomized controlled trial.. Neonatology.

[12] Bornstein MH (1989). Sensitive periods in development: Structural characteristics and casual intrepretations.. Psychol Bull.

[13] Anderson GC (1977). The mother and newborn: Mutual caregivers.. JOGN Nurs.

[14] Odent M (2001). New reasons and new ways to study birth physiology.. Int J Gynaecol Obstet.

[15] Winberg J (1995). Examining breast-feeding performance: forgotten influencing factors.. Acta Paediatr.

[16] American Academy of Pediatrics Section on Breastfeeding (2012). Breastfeeding and the use of human milk.. Pediatrics.

[17] National Population Commission (NPC) [Nigeria] and ICF International. Nigeria Demographic and Health Survey 2013. Abuja, Nigeria, and Rockville, Maryland, USA: NPC and ICF International; 2014.

[18] National Institute of Population Research and Training (NIPORT), Mitra and Associates, and ICF International. Bangladesh Demographic and Health Survey 2014. Dhaka, Bangladesh, and Rockville, Maryland, USA: NIPORT, Mitra and Associates, and ICF International; 2016.

[19] Begum K, Dewey K. Impact of early initiation on breastfeeding on neonatal deaths. A&T Technical Brief. Issue 1. FHI 360. Washington, DC: FHI 360, Alive and Thrive; 2010.

[20] Edmond KM, Zandoh C, Quigley MA, Amenga-Etego S, Owusu-Agyei S, Kirkwood BR (2006). Delayed breastfeeding initiation increases risk of neonatal mortality.. Pediatrics.

[21] Mullany LC, Katz J, Li YM, Khatry SK, LeClerq SC, Darmstadt GL (2008). Breast-feeding patterns, time to initiation, and mortality risk among newborns in southern Nepal.. J Nutr.

[22] World Health Organization, United Nations Children’s Fund. Baby-Friendly Hospital Initiative: Revised, updated, and expanded for integrated care. Geneva: WHO; 2009. 23926623

[23] American College of Obstetrics and Gynecologists Committee on Obstetrics Practice, Committee on Health Care for Underserved (2013). Special report from ACOG. Breastfeeding: Maternal and infant aspects.. ACOG Clin Rev.

[24] Sobel HL, Silvestre MAA, Mantaring JBV, Oliveros YE, Nyunt US (2011). Immediate newborn care practices delay thermoregulation and breastfeeding initiation.. Acta Paediatr.

[25] Crenshaw JT (2014). Healthy Birth Practice #6: Keep mother and baby together—it’s best for mother, baby and breastfeeding.. J Perinat Educ.

[26] Hobbs AJ, Mannion CA, McDonald SW, Brockway M, Tough SC (2016). The impact of caesarean section on breastfeeding initiation, duration and difficulties in the first four months postpartum.. BMC Pregnancy Childbirth.

[27] Rowe-Murray HJ, Fisher JRW (2002). Baby friendly hospital practices: cesarean section is a persistent barrier to early initiation of breastfeeding.. Birth.

[28] Kerber KJ, de Graft J, Bhutta Z, Okong P, Starrs A, Lawn J (2007). Continuum of care for maternal, newborn,and Child health: from slogan to service delivery.. Lancet.

[29] Campbell OMR, Calvert C, Testa A, Strehlow M, Benova L, Keyes E (2016). The scale, scope, coverage, and capability of childbirth care.. Lancet.

[30] Feldman-Winter L, Goldsmith JP, AAP Committee on Fetus and Newborn, AAP Task Force on Sudden Infant Death Syndrome (2016). Safe sleep and skin-to-skin care in the neonatal period for healthy term newborns.. Pediatrics.

[31] Berde AS, Yalcin SS (2016). Determinants of early initiation of breastfeeding in Nigeria: A population-based study using the 2013 demographic and health survey data.. BMC Pregnancy Childbirth.

[32] Sharma IK, Bryne A (2016). Early initiation of breastfeeding: A systematic literature review of factors and barriers in South Asia.. Int J Breastfeed..

[33] Esteves TMB, Daumas RP, Couto de Oliveira MI, de Ferreira de Andrade CA, Leite IC (2014). Factors associated to breastfeeding in the first hour of life: Systematic review.. Rev Saude Publica.

[34] Adhikari M, Khanal V, Karkee R, Gavidia T (2014). Factors associated with EIBF among Nepalese mothers: further analysis of Nepal Demographic and Health Survey, 2011.. Int Breastfeed J.

[35] Blanc AK, Warren C, McCarthy KJ, Kimani J, Ndwiga C (2016). RamaRao S. Assessing the validity of indicators of the quality of maternal and newborn health care in Kenya.. J Glob Health.

[36] Stanton CK, Rawlins B, Drake M, dos Anjos M, Cantor D, Chongo L (2013). Measuring coverage in MNCH: Testing the validity of women’s self-report of key maternal and newborn health interventions during the peripartum period in Mozambique.. PLoS One.

[37] Manasyan A, Chomba E, McClure EM, Wright LL, Krzywanski S, Carlo WA (2011). Cost-effectiveness of essential newborn care training in urban first-level facilities.. Pediatrics.

